# The effect of patient education based on health belief model on hospital readmission preventive behaviors and readmission rate in patients with a primary diagnosis of acute coronary syndrome: a quasi-experimental study

**DOI:** 10.1186/s12872-021-02413-8

**Published:** 2021-12-16

**Authors:** Hossein Habibzadeh, Aynaz Bagherzadi, Alireza Didarloo, Hamidreza Khalkhali

**Affiliations:** 1 Department of Medical-Surgical Nursing, School of Nursing and Midwifery, Nursing and Midwifery Faculty, Urmia University of Medical Sciences, Campus Nazlu, 11 KM Road Seru, 575611-5111 Urmia, West Azerbaijan Iran; 2 Department of Public Health, Faculty of Health Sciences, Urmia University of Medical Sciences, Campus Nazlu, 11 KM Road Seru, 575611-5111 Urmia, West Azerbaijan Iran; 3 Department of Biostatistics and Epidemiology, School of Medicine, Nursing and Midwifery Faculty, Urmia University of Medical Sciences, Campus Nazlu, 11 KM Road Seru, 575611-5111 Urmia, West Azerbaijan Iran

**Keywords:** Readmission, Health belief model, Acute coronary syndrome, Behavior, Prevention

## Abstract

**Background:**

The health belief model is one of the applicable methods of training health preventive behaviors, especially in patients with cardiovascular diseases. Therefore, this study aimed to determine the effect of patient education based on the health belief model on readmission preventive behaviors and readmission rate in patients with a primary diagnosis of acute coronary syndrome.

**Methods:**

The present quasi-experimental study was conducted in 2020 on patients with a primary diagnosis of acute coronary syndrome who were discharging from Seyed Al-Shohada Hospital, Urmia, Iran. In this study, a total of 70 samples were recruited using convenience sampling and then randomly assigned to two groups of intervention and control (*n* = 35 in each group). A total of 7 face-to-face group training sessions were held with the participation of the patients and one of their family members during 14 days after hospital discharge. These sessions were conducted along with concentration on the structures of the health belief model. Data were collected at three time points of immediately before, one month, and three months after the intervention using a demographic questionnaire, a researcher-made questionnaire of readmission preventive behaviors in cardiovascular diseases, and a checklist of hospital readmission. Data were analyzed using SPSS Statistics for Windows, version 17.0 (SPSS Inc., Chicago, Ill., USA).

**Results:**

The results showed that there was a statistically significant difference in the mean score of preventive behaviors between the two groups at time points of one month and three months after the intervention (*p* < *.05*). However, there was no statistically significant difference in the readmission rate between the two groups after the intervention (*p* > *.05*).

**Conclusion:**

Health belief model-based education was shown to be effective on readmission preventive behaviors in patients with acute coronary syndrome, although this model had no effect on the readmission rate in these patients. Other factors affecting the readmission rate are recommended to be investigated.

**Supplementary Information:**

The online version contains supplementary material available at 10.1186/s12872-021-02413-8.

## Background

According to the statistic by World Health Organization (WHO), Ischemic Heart Disease (IHD) is the leading cause of death globally [1]. Meanwhile, about 7.2 million (13%) of deaths are due to Coronary Artery Disease (CAD) and it is estimated that over 780,000 people per year experience Acute Coronary Syndrome (ACS) in the United States [2]. The incidence of CAD has increased significantly in recent years in Iran. The prevalence of cardiovascular diseases (CVDs) is about 45% in Iran so that it is significantly higher than in Western countries [3].

The per capita cost of CVD treatment in low-income and middle-income countries is 0.43–0.90$ and 0.54–2.93$, respectively [4]. CAD is the beginning point of many heart problems so that it can lead the patient to medical centers even after discharge [5]. Nowadays, the issue of hospital readmission is more important than in the past since the cost of care is rapidly increasing and multiple hospitalizations cause heavy economic costs, disrupt familial roles, distance the patient from his/her workplace, and increase the provision of healthcare services [6]. The standardized frequency of ACS readmission is 18.3% globally [7]. In Iran, the hospital readmission rate is 57% and the relative frequency of readmission in terms of disease type is 52% for IHD [8].

Prevention of CVDs through either lifestyle changes or pharmacotherapy is cost-effective. The elimination of health-threatening behaviors prevents CVDs by at least 80%. Prevention of CVDs in the general population should be achieved by promoting healthy lifestyle behaviors and individually combating unhealthy lifestyles [9]. Health preventive behavior refers to any activity that is taken by a person, who believes that the behavior is healthy, to prevent or diagnose a disease in an asymptomatic state. Behavior change is the greatest hope for reducing morbidity and avoidable mortality worldwide [10]. Nursing education can reduce hospital readmission in patients with CVDs [11]. Patient education based on Health Belief Model (HBM) can improve health preventive behaviors [12]. HBM is known as one of the health models for the prevention of health problems such as CVDs [13] and has been used in research and clinical settings [14]. HBM is a psychological model that predicts the health "behaviors" of individuals based on their beliefs and perspectives [14]. This model has the potential to be utilized in individual and collective education for preventing diseases and their complications [15].

Concerning the need for lifestyle improvement in ACS patients, the prevention of hospital readmission as a preventable ACS complication, and the lack of studies in this area, we found it necessary to conduct a study aiming to determine the effect of education based on the health belief model on readmission preventive behaviors and readmission rate in patients with a primary diagnosis of ACS. Based upon the results of previous studies in this area, the hypotheses of this study were formulated and examined as follows:

1. Patients who receive HBM-based training will perform better compared to those in the control group.
2. The readmission rate will be lower in patients who receive HBM-based training compared to those in the control group.

## Methods

### Study design and setting

This is a two-arm, single-blinded, quasi-experimental study with a pretest–posttest design conducted in 2020 at Seyed Al-Shohada Hospital, Urmia, Iran.

### Participants and sample size

In the present study, patients with a primary diagnosis of ACS constituted the target population. Regarding the mean ± SD of the intervention (3.07 ± 0.42) and the control group (3.62 ± 0.62) in a study by Zainali et al. [16] and considering the confidence interval of 0.95 and the power of 0.95, the minimum sample size was calculated to be 58 (*n* = 29 in each group) using G*Power 3.1. software [17]. Concerning the probability of 20% attrition, the final sample size [18] was considered to be 70 (*n* = 35 in each group).

$$n = \frac{{\left( {Z_{{1 - \frac{\alpha }{2}}} + Z_{1 - \beta } } \right)^{2} \left( {\delta_{1}^{2} + \delta_{2}^{2} } \right)}}{{\left( {\mu_{1} - \mu_{2} } \right)^{2} }}$$

Inclusion criteria consisted of the followings: (a) willingness to participate in the study, (b) being in the 18–65 age range, (c) having no mental health disorder, (d) having ejection fraction above 45%, (e) having no underlying comorbidities, and (f) controlled diabetes (fasting blood sugar of less than 126 mg/dl) or having no diabetes. Participants who were reluctant to continue participation in the study and were absent from more than one training session or did not complete the questionnaires were excluded from the study.

### Data collection

Data collection was conducted using a demographic questionnaire, a researcher-made questionnaire of readmission preventive behaviors in CVDs, and a checklist of hospital readmission.

The demographic questionnaire included items on age, gender, marital status, occupation, educational level, past medical history, smoking history, laboratory values (high-density lipoprotein, low-density lipoprotein, triglycerides, cholesterol), height, weight, and blood pressure.

This questionnaire was developed by the researcher and consists of 68 items in three domains of knowledge (11 items), HBM constructs (42 items including 5 items on perceived sensitivity, 7 items on perceived severity, 7 items on perceived benefits, 7 items on perceived barriers, 8 items on perceived self-efficacy, and 8 items on action guides), and CVDs preventive behaviors (15 items). Each item of the questionnaire is scored on a 5-point Likert scale (5 = Strongly Agree to 1 = Strongly Disagree). The reliability of the instrument was calculated using the internal consistency method so that the Cronbach's alpha of the domains of HBM constructs and preventive behaviors was obtained to be 0.78–0.86 and 0.92, respectively [16]. Moreover, the face and content validity of the questionnaire was assessed using the expert panel so that the questionnaire was first given to 10 professors of nursing, health, and cardiology departments and their comments were then applied to modify the items.

The checklist of hospital readmission was first developed by Rezaie et al. [19]. Items of this checklist were extracted using scientific texts and its content was approved by 10 medical and nursing faculty members of Urmia University of Medical Sciences. To evaluate the frequency of hospital readmission, the researcher completed the checklist by asking patients (through a phone call or face to face) about their history of re-hospitalization within the first and third months after discharge and the reason for it.

### Intervention

After obtaining approval from the Ethics Research Committee of Urmia University of Medical Sciences and receiving the necessary permission from the hospital authorities, the researcher introduced herself to the participants and granted written informed consent from those eligible patients who showed willingness to participate in the study. Then a total of 70 eligible patients were selected using convenience sampling during a 2-week period and randomly allocated to two groups of intervention and control (*n* = 35 in each group). The restricted randomization method was utilized to conduct randomization and remove the bias. So a total of 70 cards (n = 35 of A cards, n = 35 of B cards) were first prepared and placed in an opaque and sealed envelope. Each participant was then asked to pick up one of the cards, based on the letter of which he/she was allocated to the intervention or the control group. After the above stages, a pretest was conducted using the demographic questionnaire, the questionnaire of readmission preventive behaviors, and the checklist of hospital readmission.

The intervention group was first divided into groups of 3 patients and then each group received seven face-to-face 90-min training sessions during the first two weeks after discharge (one session every other day). These sessions were held during the evening shifts with the participation of the patients and one of their family members. Patients in the control group received no intervention. Both groups received routine post-discharge care.

Educational content was developed and prepared in form of a booklet mainly based on a book by Sharma and Romas [20] entitled "Theoretical Foundations of Health Education and Health Promotion". The researcher also utilized articles, guidelines, and up-to-date and valid books in areas of ACS and hospital readmission to prepare the content. Moreover, the educational content concentrated on the HBM constructs and consisted of three dimensions of public health education (including general health information), psychological-educational interventions (including the effective method for stopping smoking and reducing depression), and secondary prevention (including strategies to promote a healthy lifestyle, medication management, and reduce complications of CVDs). The content was also approved by 10 faculty members of Urmia School of Nursing and Midwifery and School of Health (Table 1). The training booklets were delivered to the patients after the completion of the training session. The researcher's phone number was granted to the patients in the intervention group, through which they could contact the researcher for 3 months from 8 am to 8 pm and ask their questions. The reminder text messages about lifestyle modification were sent to the patients in the intervention group every week. In the follow-up stage, the hospital readmission rate during 1 month and 3 months after discharge was evaluated in both groups (face to face and phone call) and recorded in the readmission checklist. Two post tests were conducted at time points of 1 month and 3 months after discharge using the questionnaire of preventive behaviors (see Additional file 1).

**Table 1:** The educational content of HBM sessions

Session no	Educational content
1st	Introduction, providing general information about CVDs, symptoms, diagnosis, and treatments
2nd	Playing videos about hospital readmission, salt and fat consumption, fast food consumption, and patient's physical activity, expressing the statistics of ACS and its readmission rate throughout the world, Iran, and West Azerbaijan to increase the perceived sensitivity to readmission and ACS
3rd	Providing CVDs and their readmission rate statistics, case studies, and readmission complications e.g. disruption of familial roles, distance from the workplace, and increased provision of health services, and heavy economic costs to increase perceived severity to readmission and ACS
4th	Conducting group discussion about readmission and ACS to promote perceived benefits, conducting group discussion about the advantages of ACS complying with ACS readmission preventive behaviors to moderate perceived benefits
5th	Creating brainstorming, thinking about inappropriate behaviors, introducing other wrong alternative methods, and overcoming inappropriate behaviors to improve perceived barriers
6th	Visual reminding to improve action guides e.g. sending weekly text messages to improve lifestyle and encourage the use of radio and television health broadcasts
7th	Playing a video on taking small steps to improve perceived self-efficacy, such as controlling your pulse rate and blood pressure and using a pillbox to not forget to take medication

### Data analysis

Data analysis was conducted using SPSS Statistics for Windows, version 17.0 (SPSS Inc., Chicago, Ill., USA). Kolmogorov–Smirnov test was applied to determine the normality of data distribution. In the descriptive statistics, the parameters of frequency and percentage were used for analyzing qualitative variables and the parameters of mean and standard deviation were used for analyzing normal quantitative variables. In the analytical statistics, chi-squared and Fisher's exact tests were utilized to evaluate the homogeneity of the groups. Finally, repeated measures Analysis of Variance (rANOVA) was used to conduct the within-group comparisons. A significant level was considered to be 0.05. Furthermore, data analysis was performed by an analyst who was blinded to the random allocation and data collection.

## Results

### Demographic characteristics

The results showed that there was no statistically significant difference between the two groups in terms of age, gender, marital status, occupation, educational level, past medical history, smoking history, laboratory values, height, weight, and blood pressure in (*p* > *0.05*). In other words, the two groups were homogeneous in terms of the above variables (Tables 2 and 3).

**Table 2:** Comparison of demographic characteristics between the intervention and control groups

Variable	Group				Results
	Control		Intervention		
	Frequency	Percentage (%)	Frequency	Percentage (%)	
**Gender**					
Male	17	50	17	48.6	*χ*^*2*^ = *.014* *df* = *1* *Sig* = *.906*
Female	18	50	18	51.4	
**Education**					
Illiterate	7	20	8	22.9	*χ*^*2*^ = *1.01* *df* = *4* *Sig* = *.909*
Primary	16	45.7	14	40	
Secondary	7	20	8	22.9	
Tertiary	5	14.3	5	14.3	
**Occupation**					
Employed	21	60	16	45.7	*χ*^*2*^ = *1.43* *df* = *1* *Sig* = *231/0*
Unemployed	14	40	19	54.3	
**Marital status**					
Single	5	14.3	55	14.3	*χ*^*2*^ = *.00* *df* = *1* *Sig* = *1.00*
Married	30	85.7	30	85.7	
**CVD history**					
Yes	16	45.7	17	48.6	*χ*^*2*^ = *.057* *df* = *1* *Sig* = *.811*
No	19	54.3	18	51.4	
**Hypertension history**					
Yes	23	65.7	25	71.4	*χ*^*2*^ = *.265* *df* = *1* *Sig* = *.607*
No	12	34.3	10	28.6	
**Diabetes history**					
Yes	23	65.7	25	71.4	*χ*^*2*^ = *.00* *df* = *1* *Sig* = *1.00*
No	12	34.3	10	28.6	
**Hyperlipidemia history**					
Yes	22	62.9	21	60	*χ*^2^ = *.060* *df* = *1* *Sig* = *.806*
No	13	37.1	14	40	
**Smoking history**					
Yes	13	37.1	12	34.3	*χ*^2^ = *.062* *df* = *1* *Sig* = *.803*
No	22	62.9	23	65.7

**Table 3:** Comparison of demographic characteristics between the intervention and control groups

Variable	Group				Results
	Control		Intervention		
	Mean	SD*	Mean	SD*	
Age	54.53	19.9	51.56	37.8	*t* = *−1.68* *df* = *68* *Sig* = *.096*
High blood pressure	71.139	25.20	57.135	38.23	*t* = *.792* *df* = *68* *Sig* = *.431*
Low blood pressure	29.82	78.33	00.87	3.17	*t* = *−.737* *df* = *68* *Sig* = *.464*
High-density lipoprotein	20.48	10.18	3.44	98.20	*t* = *.891* *df* = *68* *Sig* = *.376*
Low-density lipoprotein	43.113	47.40	63.109	5.53	*t* = *.420* *df* = *68* *Sig* = *.676*
Triglycerides	54.150	28.42	89.137	70.27	*t* = *1.481* *df* = *68* *Sig* = *.143*
Cholesterol	60.200	61.45	66.208	1.51	*t* = *−.697* *df* = *68* *Sig* = *.488*
Weight	56.78	78.10	57.75	71.7	*t* = *1.327* *df* = *68* *Sig* = *.189*
Height	29.167	9.7	74.164	47.8	*t* = *1.355* *df* = *68* *Sig* = *.180*

^*^Standard Deviation

### The readmission preventive behavior

The rANOVA was used to investigate the effect of HBM-based patient education on the readmission preventive behaviors at three time points of before, one month after, and three months after the intervention. The results of rANOVA indicated that there was a statistically significant difference in the mean score of readmission preventive behaviors between the time points of before, one month after, and three months after the intervention in the intervention group (*p* < *0.001*). However, this difference was not shown to be statistically significant different (*p* = *0.142*) (Table 4). Therefore, the first hypothesis of the study is accepted.

**Table 4:** Mean, standard deviation, and the results of ANOVA regarding the overall mean scores of readmission preventive behaviors

Overall score	Mean and SD* of overall scores at three measurement time points						*Sig*	*Partial Eta Squared*
Group	Before the intervention		One month after the intervention		Three months after the intervention			
	Mean	SD*	Mean	SD*	Mean	SD*		
Control	143.11	17.33	138.71	19.27	136.17	20.07	*.142*	*.031*
Intervention	137.23	17.39	151.77	19.70	148.40	20.27	*.000*	*.033*

The results of binary comparisons based on Bonferroni correction showed no statistically significant difference in the mean score of readmission preventive behaviors between the two groups before the intervention (*p* = *0.410*). However, this difference was found to be significantly different between the two groups at time points of one month and three months after the intervention (*p* < *0.001*) (Table 5).

**Table 5:** Results of binary comparisons based on Bonferroni correction between the intervention and control groups at three measurement time points

Measurement time point	Group	Mean difference	SD difference	*p*-value
Before the intervention	Intervention-Control	1.40	1.69	*.410*
One month after the intervention	Intervention-Control	*−*7.97	1.90	*.000*
Three month after the intervention	Intervention-Control	*−*7.31	1.96	*.000*

The results of binary comparisons based on Bonferroni correction also showed a statistically significant difference between the overall mean scores of readmission preventive behaviors at time points of before and after the intervention (one month and three months after) in the control group (*p* < *0.05*) (Table 5).

The results of binary comparisons based on Bonferroni correction also showed a statistically significant difference between the overall mean scores of readmission preventive behaviors at time points of before and after the intervention (both one month and three months after) in the control group (*p* < *0.05*). This difference was also shown to be statistically significant between the overall mean scores at time points of one month and three months after the intervention in the control group (*p* < *0.05*). All of the above differences were found to be statistically significant in the intervention group (*p* < *0.05*). The results showed that the mean scores of readmission preventive behaviors decreased significantly in the control group, while these mean scores increased significantly in the intervention group at all of the three measurement time points. This indicated a positive effect of the HBM-based educational intervention on patients' readmission rate and preventive behavior (Table 6).

**Table 6:** Within-group comparison of the overall mean scores in the intervention and control groups

Group	Difference between measurement time points	Mean difference	SD difference	*p*-value
Control	Before the intervention-one month after the intervention	4.40	1.74	*.041*
	Before the intervention-three months after the intervention	6.94	1.97	*.002*
	One month after the intervention-three months after the intervention	2.54	.98	*.034*
Intervention	Before the intervention-one month after the intervention	*−*14.54	1.74	*.000*
	Before the intervention-three months after the intervention	*−*11.17	1.97	*.000*
	One month after the intervention-three months after the intervention	3.37	.98	*.003*

### Hospital readmission rate

The chi-square test was used to compare the hospital readmission rate between the two groups at two time points of one month and three months after the intervention. The results showed that there is no significant difference in the hospital readmission rate between the two groups at none of the above time points (*p* > *0.05*) (Table 7). Therefore, the second hypothesis of the study is rejected.

**Table 7:** Comparison of the frequency of hospital readmission between the intervention and control groups at time points of one month and three months after the intervention

Variable	Group				Results
	Control		Intervention		
	Frequency	Percentage (%)	Frequency	Percentage (%)	
Readmission one month after the intervention					
No	24	68.6	30	85.7	*χ*^*2*^ = *2.91* *df* = *1* *Sig* = *.088*
Yes	11	31.4	5	14.3	
Readmission three months after the intervention					
No	21	60	24	68.6	*χ*^*2*^ = *.56* *df* = *1* *Sig* = *.454*
Yes	14	40	11	31.4	

## Discussion

The present study was aimed at determining the effect of patient education based on the HBM on readmission preventive behaviors and readmission rate in patients with a primary diagnosis of acute coronary syndrome. The findings of the present study showed that there was no significant difference between the two groups in terms of demographic characteristics, which can affect the results. Therefore, it can be stated that the existence of a significant difference between the mean scores of dependent variables in the intervention group at time points of before and after the intervention had been due to the positive effect of HBM-based patient education. The results of this study revealed that HBM-based patient education is positively effective on the readmission preventive behaviors, although it did not affect the readmission rate in ACS patients. The ineffectiveness of this model on the readmission rate could be due to the small sample size of the study. Therefore, it can be concluded that behavior change is not the only effective factor in reducing the readmission rate and there are other factors that need to be identified and adjusted using educational models.

In line with the results of our study, Kheiri et al. [21] showed that the implementation of HBM can promote health preventive behaviors among patients with CVDs. Vahedian-Shahroodi et al. [22] also indicated that HBM-based nutrition education has a positive effect on students' nutritional behavior and the perceived sensitivity has the greatest impact on it among predictor variables. In the present study, HBM-based educational intervention was found to be effective in improving readmission preventive behaviors e.g. nutritional behaviors and adherence to low-salt, low-fat, and low-calorie diets. Mohammadi et al. [23] concluded that the HBM-based empowerment program increases the level of activity of daily living in ACS patients. The findings of this study also indicated that HBM-based patient education promotes CVD prevention behaviors, including nutritional behaviors, physical activity behaviors, and smoking cessation behaviors. In line with the results of our study, Amraei et al. [24] demonstrated that the HBM-oriented educational program can be used to increase nurses' preventive behaviors against CVDs and modify their diet.

Eshah [25] came to this result that nurses should be qualified to provide the necessary patient education and health education should be continued as one of the most important and common nursing care. Accordingly, the above points should be included in the discharge plan of ACS patients. In a clinical trial by Abedi et al. [26], it was shown that the application of the continuous consultation care model affects the frequency, progression, and recurrence of chest pain, so that the use of this model is effective in preventing hospital readmission. This result was inconsistent with the results of our study, which could be due to differences in the educational pattern or a longer educational period (six months) in their study.

In line with the results of our study, Rouhani et al. [27] showed that patient education based on learning needs is not effective on the hospital readmission rate in patients with heart failure. Ho et al. [28] showed that the educational intervention was effective in promoting awareness and self-care behaviors and reducing the readmission rate of patients with heart failure. Therefore, hospital readmission can be minimized by developing proper health plans and providing proper patient education aimed at self-care improvement.

### Limitations

One of the limitations of this study was the occurrence of the COVID-19 pandemic during the intervention time period. Concerning the outbreak of coronavirus and the lack of samples in cardiology hospitals, sampling was limited to only one hospital. Another limitation of the study is the number of people studied in each intervention group. The number of people in each intervention group was restricted to three people (intervener, patient, and one patient's family member). Moreover, due to the peak outbreak of coronavirus and lockdown three months after the intervention, patients were not satisfied to come to the hospital to complete the questionnaires, so that the questionnaires were completed by calling the patients. The small sample size was another limitation of the study. Therefore, other studies should be conducted with larger sample sizes to obtain more accurate results. One of the other limitations of this study was to evaluate the readmission rates in a short term, so it is recommended that similar studies should be conducted on larger populations over a longer time period.

## Conclusion

The HBM-based patient education was indicated to be effective in promoting readmission preventive behaviors. However, this model did not affect the hospital readmission rate among ACS patients. Regarding what mentioned, other factors affecting hospital readmission (e.g. early discharge, poor patient education, poor health staff education, poor symptom control) are recommended to be investigated and identified in qualitative studies.

## Supplementary Information

**Additional file 1**. TREND Statement Checklist.

## Data Availability

All data generated or analysed during this study are included in this published article. And any additional data/files may be obtained from the corresponding author on reasonable request**.**

## Abbreviations

WHO: World Health Organization
IHD: Ischemic Heart Disease
CAD: Coronary Artery Disease
ACS: Acute Coronary Syndrome
HBM: Health Belief Model
